# Label-Free QCM Immunosensor for the Detection of Ochratoxin A

**DOI:** 10.3390/s18041161

**Published:** 2018-04-11

**Authors:** Şerife Şeyda Pirinçci, Özlem Ertekin, Duygu Ercan Laguna, Fehime Şeyma Özen, Zafer Ziya Öztürk, Selma Öztürk

**Affiliations:** 1 TÜBİTAK, The Scientific and Technological Research Council of Turkey, Marmara Research Center, Genetic Engineering and Biotechnology Institute, Gebze, Kocaeli 41400, Turkey; duygu.ercan@windowslive.com (D.E.L.); f.seymaozen@gmail.com (F.Ş.Ö.); selma.ozturk@tubitak.gov.tr (S.Ö.); 2 Department of Medical Genetics and Molecular Biology, Kocaeli University, Umuttepe, Kocaeli 41100, Turkey; 3 Department of Physics, Gebze Technical University, Gebze, Kocaeli 41400, Turkey; zozturk@gtu.edu.tr

**Keywords:** OTA, Ochratoxin A, QCM, quartz crystal microbalance, immunobiosensor, regeneration, immobilization

## Abstract

Ochratoxin A (OTA) is a potent mycotoxin that poses a risk in food and feed moieties and subject to worldwide regulation. Laboratory-based analytical methods are traditionally employed for reliable OTA quantification, but these methods cannot provide rapid and on-site analysis, where biosensors fill this gap. In this study a label-free quartz crystal microbalance (QCM)-based immunosensor for the detection of OTA, which is one of the most important small molecule contaminants, was developed by direct immobilization of OTA to amine-bearing sensor surfaces using 1-ethyl-3-(3-dimethylaminopropyl) carbodiimide (EDC)/N-Hydroxysuccinimide (NHS) chemistry. The protein-free sensor surface enabled regeneration of sensor surface with 50 mM NaOH and 1% SDS up to 13 times without loss of performance, which would disrupt a protein-containing sensor surface. We developed a QCM immunosensor using the developed sensor surface with a 17.2–200 ng/mL detection range which can be used for on-site detection of feedstuffs.

## 1. Introduction

Ochratoxin A (OTA) is one of the most dangerous and common mycotoxins produced by *A. ochraceus*, *A. carbonarius*, *A. niger* and *P. verrucosum* species, and affect human and animal health. OTA is known to be carcinogenic, nephrotoxic, hepatotoxic, neurotoxic, teratogenic, and immunotoxic. It induces kidney cancer in animals and is considered as Group 2B possible human carcinogen [1,2,3,4]. Other side effects of OTA are the inhibition of macromolecule synthesis, an increase in lipid peroxidation, and inhibition of mitochondrial respiration [5,6,7]. In addition, OTA is associated Balkan Endemic Nephropathy (BEN) and chronic interstitial nephropathy (CIN) [8,9,10,11].

OTA poses a risk factor for a wide variety of food and feed products including cereals, dried fruits, wine, and coffee [12,13]. Among these products, cereals are the most commonly contaminated commodity with OTA and constitute up to 80% of swine, poultry, and pig diets [14,15]. OTA’s high affinity to proteins and increased stability when bound to proteins results in the accumulation of OTA in organs of animals, which leads to transmission of OTA by consumption of products of animal origin [16]. Moreover, OTA-contaminated feed not only affects human health through the food chain, but also reduces animal growth rates and impacts productivity, especially in pork and poultry production [15]. 

Due to its carcinogenic, nephrotoxic, hepatotoxic, neurotoxic, teratogenic, and immunotoxic effects, as well as direct impact on animal husbandry, OTA content in food and feed products is regulated [17,18]. Although the laboratory-based methods used for the quantification of OTA, such as LC MS, GC and HPLC, provide quite sensitive and reliable results, these methods are time-consuming, expensive, and require a trained operator [19]. In addition to chromatographic methods, immunoassays, such as ELISA, EIA, and RIA can be used in the detection of OTA. Although these immunological methods are cheaper and easier than chromatographic methods, the need for long incubation times for detection of low concentration analytes in the stationary phase and involvement of many steps prevent the easy and wide use of these methods in the field. 

Studies show that despite all monitoring efforts and regulations, the presence of mycotoxin contamination in feedstuff cannot be prevented properly. In a striking study conducted by Rodrigues and Naehrer, it was shown that 81% of 7049 feed samples collected from Asia, Europe, and America contain mycotoxins of which 48% was contaminated with more than one mycotoxin [20]. This may be due to the fact that mycotoxin contamination may arise or increase during storage, processing, handling, or even marketing of feed after mycotoxin analysis. At this point, biosensors can help reduce the presence rate of mycotoxins by enabling fast, easy, cheap, sensitive, specific, on-site, and frequent analysis of samples [21,22,23]. 

QCM transducers are employed for the development of biosensors for many biological analytes due to their high sensitivity without labels [24]. In QCM systems, quartz crystals with piezoelectric properties are employed. The AT-cut quartz crystals used in QCM transducers show piezoelectric properties and resonate at a fixed frequency upon application of an electric current [25]. This frequency changes by changing the conditions in contact with the crystal. The conditions which change the readout frequency were defined with Sauerbrey’s equation [26]: (ΔF = −2F_0_^2^Δm/A(ρ_q_μ_q_)^1/2^), where ΔF is the counted frequency change (Hz); F_0_ is the fundamental resonance frequency of the quartz oscillator; Δm is the mass change; A is the area of the electrode; ρ_q_ is quartz density; and μ_q_ is the shear stress of quartz. As can be seen from the equation, mass change on the surface of the quartz crystal is directly proportional to the frequency change and, hence, the mass deposit on the crystal surface can be used for biosensing applications. When the surface is functionalized for specific detection of an analyte, the binding of the recognition element can be observed in real-time with an appropriate frequency reader [24]. QCM is widely used in the detection of various analytes in low concentrations, such as bacteria [27], mycotoxins [28,29], disease markers [30], viruses [31], and many other analytes due to its simplicity, low cost, and sensitivity [32]. Another advantage of QCM over other endpoint measuring tools is its ability to make real-time measurements. This feature provide it the potential of usage in automated continuous monitoring systems [33]. Making real-time measurements also makes it a suitable system for characterization of affinity-based systems, surfaces, and recognition elements [32]. 

Among different assay systems, immunoassays utilizing the specificity and selectivity of antibodies as recognition elements are widely used. When the target analytes are small molecular weight compounds such as mycotoxins, the change created by binding of the analyte to a functionalized sensing area cannot be detected. Mycotoxins also do not allow the use of sandwich immunoassay systems since these molecules have only one epitope. Thus, in this case, a competitive immunoassay format is employed rather than a direct immunoassay [34,35]. Two different competitive immunoassay formats were used for detection of OTA where either antibody or antigen was immobilized. In the antibody-immobilized system, an OTA-protein conjugate is mixed with the sample to be tested and presented to the antibody-bound surface. In a clean sample, all OTA-protein conjugates bind to the immobilized antibodies and a high frequency shift is observed. If the sample is contaminated with OTA, some of the OTA-protein conjugates will be replaced by small molecular weight OTA and a smaller frequency change will be observed. With this system, a 50–1000 ng/mL detection range can be obtained and this is the only study that immobilized antibodies to detect OTA with a QCM system [28]. This method has been used with other transducers, such as optical [36,37,38] and electrochemical systems [39,40,41]. Analyte immobilized competitive immunoassays are more frequently used for OTA analysis [42,43,44,45,46]. In these assays, OTA is immobilized to the surface and the recognition element, in this case the antibody, is delivered to the sensor surface. The specific antibody binds to the surface coated with OTA and this binding causes a frequency change. In this case OTA sensing is achieved by mixing the test sample with the antibody solution. OTA in the solution will bind to the antibody and will prevent it from binding to the OTA-immobilized sensor surface. Hence, a lower frequency change will be observed when the test sample contains OTA. In these studies, protein conjugates of OTA were used for sensor surface preparation [42,43,44]. However, the use of protein on the sensor surface may be challenging, especially when the used antibody requires denaturing conditions for regeneration, which is a case observed for hydrophobic analytes like mycotoxins [29]. Direct covalent immobilization of OTA to the sensor surface without the use of proteins confers the sensor surface stability and durability when exposed to harsh regeneration solutions [29]. In addition this strategy may increase the shelf life of the sensor surface. In this study, a method for preparation of a protein-free sensor surface for detection of carboxyl containing small analytes has been developed. This method can be successfully applied for other carboxyl-containing small analytes.

One specific example was seen in the work of Lates et al., where Ochratoxin B (OTB) was immobilized to the surface in a displacement immunoassay [47] and, to the knowledge of the authors, no OTA-immobilized surfaces were reported in the literature. 

Direct OTA immobilization is particularly challenging due to the low solubility of OTA in aqueous solutions (0.4246 mg/L) which was the reason why Lates et al. used OTB for immobilization, which is readily soluble in water (4.40 mg/L) [48]. Despite QCM systems being cheaper label-free alternatives for immunosensing, there are a limited number of works published for OTA detection by using QCM technology, and to the authors’ knowledge, no biosensor development work was conducted by direct immobilization of OTA to the sensor surface. This work was aimed to develop a QCM immunosensor employing a direct competitive immunoassay with the use of a protein-free, OTA immobilized sensor chip as well as a direct covalent immobilization method for protein-free immobilization of small analytes containing a carboxyl group. 

## 2. Materials and Methods 

### 2.1. Materials

All chemical reagents except 1-ethyl-3-(3-dimethylaminopropyl) carbodiimide (EDC) (Thermo Scientific, Waltham, MA, USA) were purchased from Sigma Aldrich, Taufkirchen, Germany. The 5 MHz AT cut quartz crystals were purchased from KVG Quartz Crystal Technology GmbH, Neckarbischofsheim, Germany. The antibody used in the immunoassays as the recognition element (10F4) was developed in-house by our laboratory and was also successfully utilized for the development of immunoaffinity columns. 

### 2.2. Preparation of the OTA Immobilized Sensor Surface

**Figure 1:**
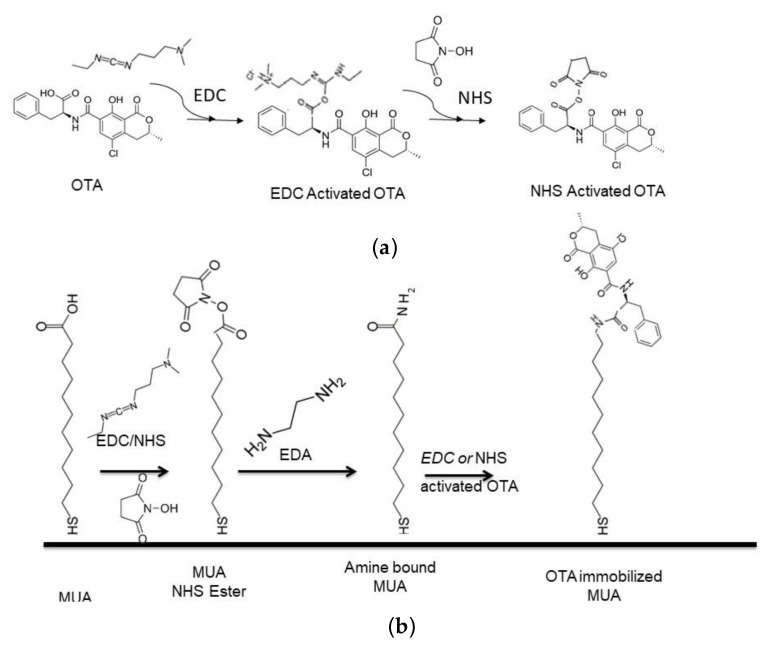
Schematic illustration of OTA immobilization to sensor surface. (**a**) OTA activation with EDC/NHS; and (**b**) immobilization of activated OTA to SAM coated sensor surface.

In this work, OTA was directly immobilized to a 11-mercaptoundecanoic acid (MUA)- bearing gold surface in order to produce a protein-free sensor chip for OTA quantification. The proposed reaction scheme consisted of activation of carboxyl group on OTA with EDC/NHS (Figure 1a) and crosslink the activated carboxy groups to the amine groups generated on MUA-coated gold sensor surface (Figure 1b). 

#### 2.2.1. Preparation of the Gold Surface for OTA Binding

Gold-coated quartz crystals were washed with ultra-pure dH_2_O prior to plasma cleaning and dried under nitrogen gas. Before surface modification, crystals were cleaned by using a Diener Femto plasma cleaner (Ebhausen, Germany) for 3 min at 40 mV. Cleaned crystals were incubated in the ethanol solution of 2 mM MUA overnight at room temperature. Then the surface coated with MUA was activated with 200 mM EDC, 50 mM NHS solution for 10 min After activation, carboxylic acid terminal moieties of MUA were converted to amine groups by incubating with 1 M ethylenediamine (EDA), pH:8.5 for 7 min free carboxylic groups were blocked with 1 M ethanolamine (EOA), pH 8.5, for 2 min.

#### 2.2.2. OTA Immobilization

For OTA immobilization to sensor surface, OTA was dissolved in ethanol in 10 mM concentration. Carboxyl groups of OTA were activated with EDC for 10 min at room temperature for its conjugation to amine moieties previously presented on quartz crystal surface. Molar ratio of EDC to OTA, ethanol concentration and final OTA concentration in different reactions are presented in Table 1. After activation, reaction mixtures were incubated on the amine functionalized surface for 15 min and OTA binding to sensor surface was achieved. Remaining free amine groups on the surface were blocked with 1 M acetate buffer, pH 4.8, for 10 min 

### 2.3. OTA Measurement Procedure

OTA-specific 10F4 antibody was used as recognition element in all measurement procedures. 0, 10, 50, 100, 200, and 500 ng/mL OTA were mixed with 0.025, 0.05, or 0.1 mg/mL 10F4 and applied to the sensor surface at room temperature with a 50 µL/min flow rate. 

Limit of detection (LOD) and limit of quantification (LOQ) for the developed sensor were calculated with the formula Limit = (k × se)/m, where k = 3.3 for LOD and k = 10 for LOQ calculations, se is the standard error for the line of best fit, and m is the slope of the linear response curve.

### 2.4. Surface Regeneration

For removal of 10F4 antibody from OTA immobilized quartz crystal surface 6 M guanidine hydrochloride, 50% methanol, 100 mM NaOH and 70 mM EDTA, 50 mM NaOH and 35 mM EDTA, 0.1 M NaOH and 0.5 M NaCl, 50 mM NaOH, 0.1 M glycine at pH 2.7, 50 mM NaOH and 1% SDS, and 0.1 M HCl were applied to the sensor surface for 2 min. Regeneration efficiencies were calculated by using the frequency reached after washing the surface with a constant PBS flow until a steady signal was achieved. 

### 2.5. Safety Considerations

OTA is a class-2B possible human carcinogen. All experiments were conducted with proper protection. All wastes containing OTA were incubated overnight in 1% hypochloride solution and sent for incineration. 

## 3. Results

### 3.1. OTA Immobilization

**Figure 2:**
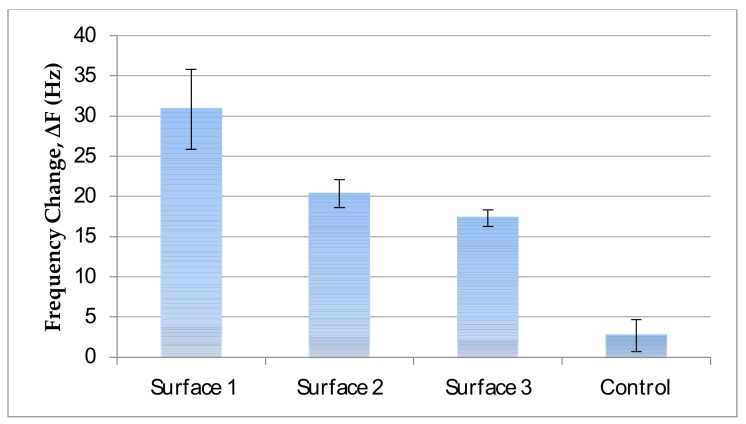
Evaluation of different OTA-immobilized surfaces for their antibody binding capacities. In control bar, BSA was sent to the surface and non-specific adsorption of the surface was measured. Error bars represent standard errors.

OTA is an ethanol soluble mycotoxin with low solubility in aqueous solutions (0.4246 mg/L) [49]. Thus, it tends to precipitate at solutions with low ethanol concentration. On the other hand, EDC and NHS, which are used for the conjugation of OTA to the amine-bearing surface precipitate at high ethanol concentrations. As such, the ethanol concentration and the concentrations of EDC/NHS and OTA should be carefully chosen so that OTA, EDC, and NHS will be soluble and active. Otherwise visible precipitates occur in the activation solution. Next, the reaction conditions that did not yield visible precipitates were evaluated for their antibody binding capacities. The frequency changes upon antibody binding to the evaluated reaction conditions in Table 1 are presented in Figure 2. The optimal conditions enabling both highest frequency change due to antibody binding to sensor surface was achieved in Surface 1 with 66% ethanol and 6.6 mM OTA concentration.

### 3.2. Regeneration

In immunobiosensor studies, the nature and strength of antigen-antibody interaction strongly determine the regeneration and reusability of the sensor surface since one needs to overcome the attractive force between the antigen and antibody to detach the binding material [50]. For this reason, regeneration conditions must be determined and optimized separately for every antigen-antibody couple. 

**Figure 3:**
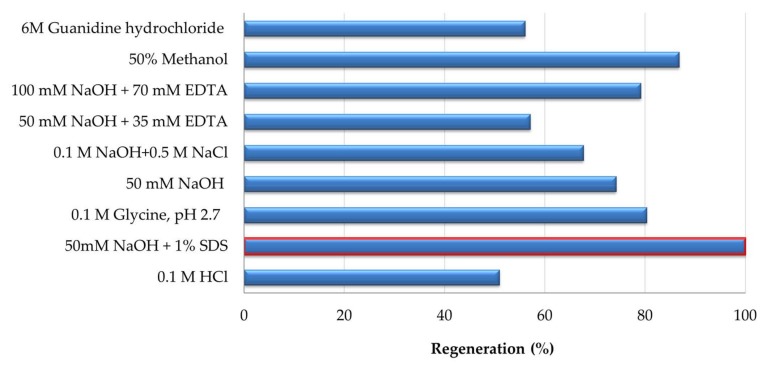
Regeneration of OTA immobilized sensor surface by using different regeneration solutions. The regeneration solution which provided 100% regeneration efficiency (50 mM NaOH and 1% SDS) was highlighted with the red border.

The surface with the highest antibody binding capacity was used for the optimization of regeneration conditions. As elucidated in Figure 3, it was only possible to fully regenerate the surface by applying 50 mM NaOH and 1% SDS, a high ionic strength detergent. Regeneration solution which could remove 10F4 antibody from the OTA-immobilized sensor surface also gives an idea about the nature of 10F4-OTA binding where the requirement of a detergent in the regeneration solution indicates the hydrophobic interaction between OTA and 10F4. The effectiveness of methanol after 50 mM NaOH and 1% SDS also corroborates this conclusion. 

**Figure 4:**
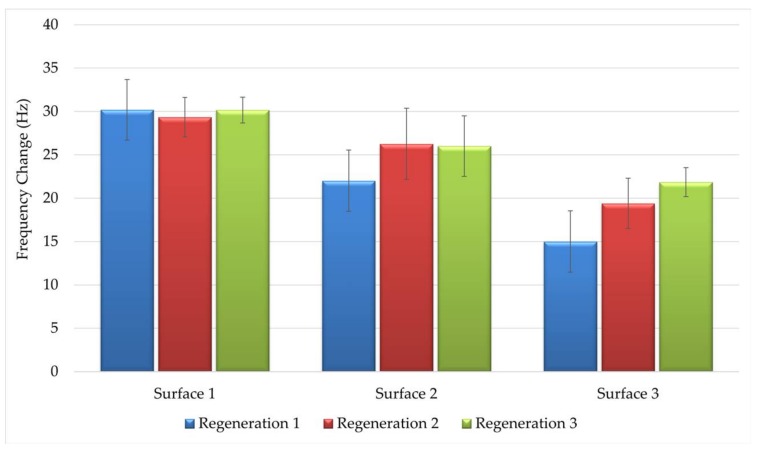
Regeneration of different OTA-immobilized sensor surfaces with 50 mM NaOH and 1% SDS regeneration solution. Error bars represent standard errors. Each experiment was repeated three times.

Reusability of the sensor surface without loss of performance is an important criterion in regeneration solution choice. A regeneration solution that renders the sensor surface functionless cannot be used even if it removes the antibody from the sensor surface with 100% efficiency. The use of detergent in the regeneration solution once again highlighted the importance of a protein-free sensor surface, where proteins on the surface would be denatured with 1% SDS in the regeneration solution if protein conjugates of OTA were to be immobilized. Thus, the regeneration efficacy depends on both the regeneration solution and the surface. As such, the surfaces indicated in Table 1 were also evaluated for their reproducibility under the harsh regeneration conditions required for the 10F4 monoclonal antibody so that the most stable surface will be used for the generation of a standard curve. The results showed that Surface 3 not only resulted in the lowest antibody binding, but also was affected by the regeneration solution and a gradual increase was observed with sequential regenerations (Figure 4). This indicates that the surface was disturbed by the regeneration solution resulting in increased non-specific protein binding. Surfaces 1 and 2 were stable with sequential regenerations (Figure 4). Surface 1, which provided the highest antibody binding and high stability in sequential regeneration was selected and further tested to determine the reusability of the regenerated surface and maximum number of regenerations in which the sensor surface is stable.

**Figure 5:**
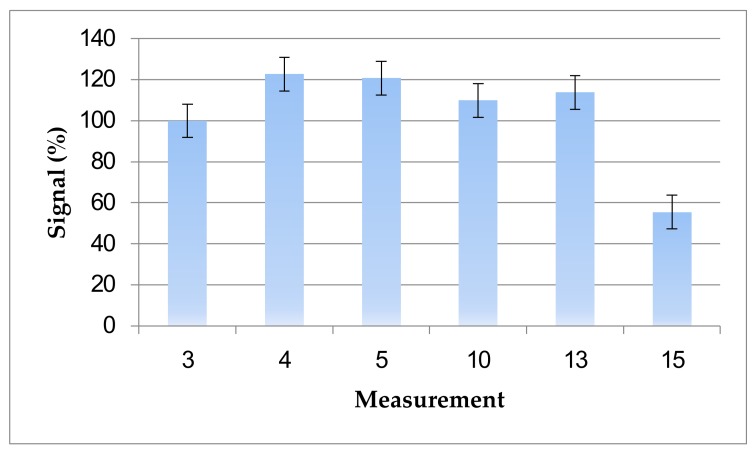
Reusability of the OTA-immobilized sensor surface after regeneration by 50 mM NaOH and 1% SDS. Measurements were made by application of 10F4 antibody to sensor surface. Error bars represent standard error. Each experiment was repeated three times.

In order to assess the durability of the sensor surface after regeneration with 50 mM NaOH and 1% SDS, repetitive measurements were performed. Results show that the surface could be regenerated up to 13 times without loss of performance (Figure 5). Higher regeneration cycles could have been achieved with milder regeneration solutions, however, the hydrophobic nature and strength of OTA-10F4 binding did not allow the use of milder conditions. 

The numbers presented in Figure 5 show the measurements made without addition of OTA in the sample. In the 6th, 7th, 8th, 9th, 11th, and 12th measurements, free OTA was mixed with anti-OTA antibody and sent to the surface. Hence, the signals measured were decreased and the results obtained by addition of OTA do not represent the regeneration efficiency and were found to eb irrelevant for this graph.

In biosensor studies, accessibility of the analyte immobilized on the sensor surface may be restrained due to the size and the density of the analyte. In this study, the possibility of losing sensitivity due to steric hindrance is evaluated by immobilization of OTA-cBSA to the sensor surface. In this experiment it was seen that although the results were comparable at the beginning, the surface prepared by immobilization of OTA-BSA quickly lost its efficiency upon regeneration with 50 mM NaOH and 1% SDS (Supplementary Materials). 

### 3.3. Assay Optimization

**Figure 6:**
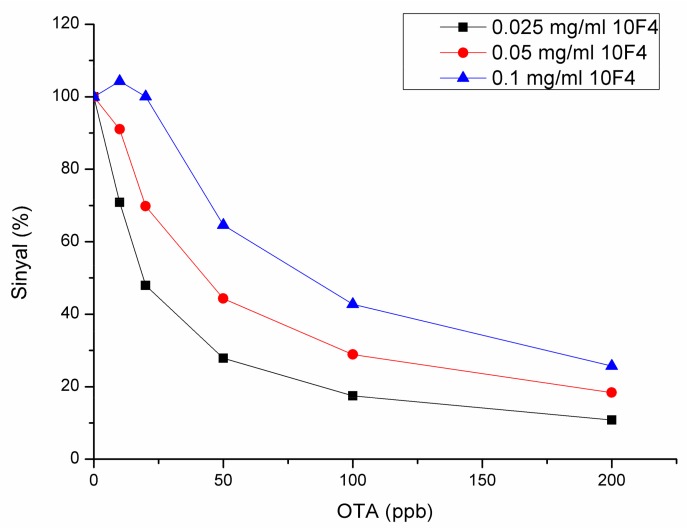
Selection of optimal antibody concentration for OTA measurement in a competitive assay format.

Most of the small molecules, like OTA, possess only one epitope and do not allow binding of more than one antibody due to their small molecular size. Therefore, these molecules are not suitable for sandwich assay format and are generally detected by using competitive assay. Competitive assay employed in this work is based on the competition of immobilized antigen and free antigen for the antibody in solution. It is well known that, in competitive systems, the assay sensitivity increases as antibody concentration decreases. The presence of excess antibody in the solution results in the need for more antigen in order to create a measurable difference in the signal. Thus, antibody concentration is one of the key parameters determining the sensitivity and limit of detection (LOD) of the assay and the surface should be prepared to be responsive to lower concentrations of the antibody. For this reason, there have been attempts to increase the binding capacity of the surface using protein conjugates, such as an SPR sensor development work where OTA was either directly conjugated to BSA or PEG was used as a linker in the conjugation reaction. The work showed that the sensitivity of the PEG-linked OTA-BSA conjugate was higher than direct OTA-BSA conjugation because the PEG-linked surface required less initial antibody concentration for efficient analysis [49]. Keeping this in mind, antibody concentration was optimized studiously. Amounts of 0.025 mg/mL, 0.05 mg/mL, and 0.1 mg/mL 10F4 were used for the determination of optimal antibody concentrations to be used in competitive immunoassay. Application of 10F4 in concentrations lower than 0.025 mg/mL did not result in a detectable frequency change (ΔF). As expected, sensitivity increased with decreasing antibody concentration (Figure 6) and 0.025 mg/mL 10F4 was selected for further measurements. 

**Figure 7:**
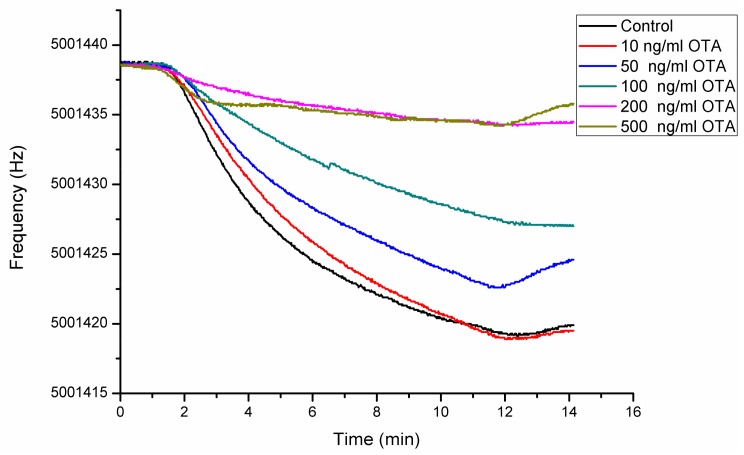
Sensorgrams showing sensor response at different OTA concentrations: 0.025 mg/mL 10F4 was applied to sensor surface until the sensorgram reaches to saturation, then the surface was washed with PBS.

Competitive assay was conducted using with 10 ng/mL–500 ng/mL OTA and the sensorgrams showing the frequency changes with changing OTA concentrations is presented in Figure 7.

**Figure 8:**
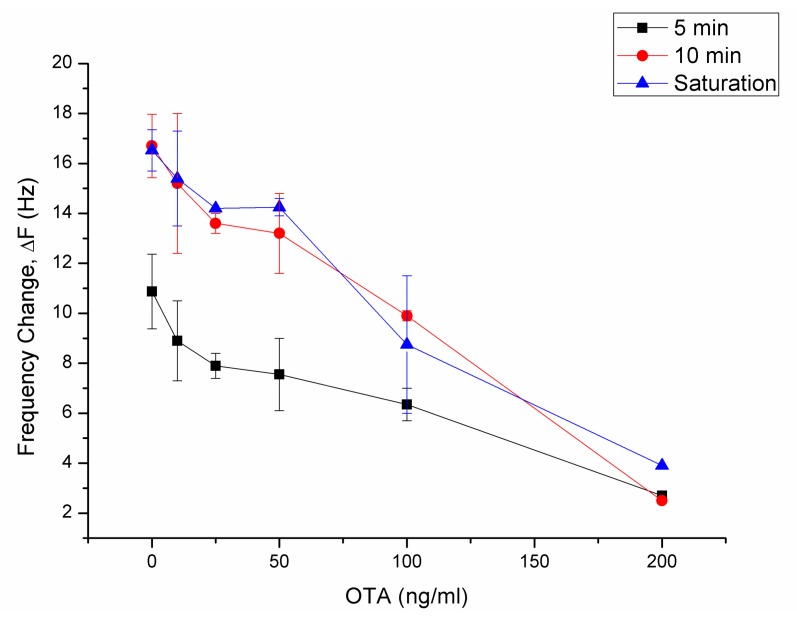
Dose-response curves for OTA at different measurement time points.

Short analysis time is one of the desired features of a diagnostic system. Thus, dose-response curves were plotted at three different time points of the sensorgrams in Figure 7 to determine the shortest possible analysis time and the time point where most sensitive measurements can be made (Figure 8). The time points selected for evaluation were 5 min and 10 min after antibody application or until a stable baseline is reached after antibody application. The frequency changes at these time points were used for the evaluation of correlation in the linear range (R^2^), LOD, and limit of quantification (LOQ) (Table 2).

Linear ranges of all three curves were between 50 ng/mL and 200 ng/mL OTA. R^2^ values, LOD, and LOQ values calculated by considering the frequency shifts at 5 min, 10 min, and saturation points are shown in Table 2. 

The measurements at saturation resulted in the lowest correlation and highest LOD and LOQ values. The saturation was reached at very different time points of the sensorgram at different concentrations. The nonstandard measurement time points decreased the sensitivity and reliability of the sensor if the saturation frequency change is considered for evaluation. A LOD of 5 min and 10 min measurements were 19.3 ng/mL and 17.2 ng/mL; a LOQ of 5 min and 10 min measurements were 58.5 ng/mL and 52.0 ng/mL respectively. Although the linear range did not differ between 5 min, 10 min, and until saturation, calculations show that the selected time point to measure the sensor response strongly affected the LOD and LOQ of OTA (Figure 8, Table 2). 

**Figure 9:**
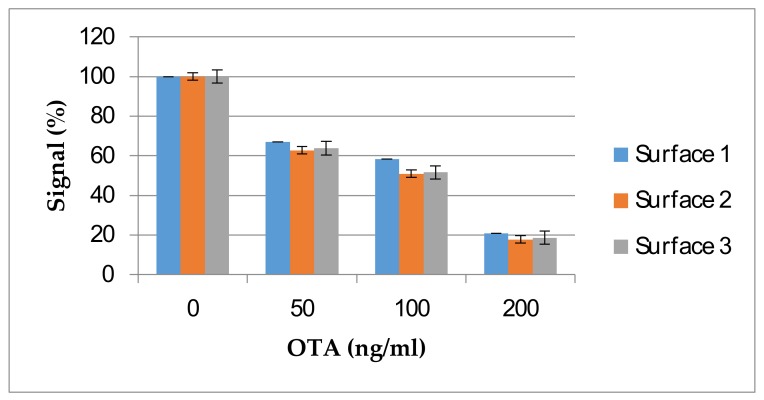
Repeatability of different sensors in terms of percent signal. Error bars represent standard errors.

The most important evaluation parameters of a sensor surface is its repeatability and consistency. In this study it was seen that the frequency change upon addition of 10F4 antibody shows high variability in each immobilization batch. However, the percent signal change upon addition of free toxin is quite reproducible and shown in Figure 9.

By optimization of the immobilization reaction conditions, regeneration solution, antibody concentration, and analysis time a QCM biosensor for detection of OTA in the 17.2–200 ng/mL range and quantification of OTA in the 52.0–200.0 ng/mL range was successfully developed. 

## 4. Discussion

In the presented work, an OTA-sensitive QCM sensor was developed by direct immobilization of OTA to the sensor surface. The immobilization of OTA to the gold-coated QCM crystal and obtaining a reproducible and stable sensing surface was the initial challenge of the work. The gold sensor surface was functionalized to bear amine groups using modified 11-MUA SAM so that the amine groups will be condensed with the carboxyl group of OTA using the well-known EDC/NHS chemistry. However, the reaction is an aqueous reaction where the most important components (EDC and NHS) are highly water soluble and the target molecule, OTA, has limited solubility in water. Even though the requirement for direct OTA immobilization to amine-bearing surfaces occurred before, the challenge could not be solved and the authors of a previous study immobilized a more soluble form of ochratoxin, OTB, instead of OTA [45]. Other work conducted to develop OTA sensors using antigen immobilized competitive immunoassays used protein conjugates of OTA in electrochemical [51,52], SPR [49], or QCM [42,43,44] transducer systems. To the authors’ knowledge, this work is the first elucidation of direct immobilization of OTA to amine-bearing surfaces. 

QCM transducer systems are preferred since a low-cost frequency reader can be used for the readouts, the system does not require prior labeling of antibodies, there are a variety of applications [53,25], and there are various QCM sensors developed for mycotoxin detection [54,55,56]. However, there are a limited number of QCM sensors for OTA analysis, where one used antibody immobilized competitive immunoassay [28] and the others used OTA-protein conjugates for immobilization [43,44,45]. Of these works, only the work of Tsai et al., which used the antibody immobilized system, utilized a simple QCM transducer, and achieved a 50–1000 ng/mL detection range [28]. The others used hybrid transducers taking advantage of two different systems. Cheap et al. used electrochemical impedance spectroscopy (EIS) coupled with a QCM and achieved a 0.1–1 ng/mL detection range, which is highly sensitive, however, not useful for food analysis since it has a very narrow and out of target detection range. Vidal and co-workers used polyclonal OTA antibodies to develop an E-QCM sensing system and achieved a 10–128 ng/mL detection range with a 30 min analysis time. They used their surface with eight possible regeneration cycles using a protease enzyme, pepsin. The reusability of their sensors would be increased if they used a protein-free surface since they used a protease for regeneration. The final QCM-based sensor we encountered in the literature was a recent work of Karczmarczyk et al. in 2007 which used a QCM-D transducer system. They achieved a linear range of 0.2–40 ng using an indirect competitive immunoassay where gold-conjugated secondary antibodies were used for signal enhancement. The total analysis time was 40 min with 15 min preincubation of the samples. The OTA sensor developed in this study used a cost-effective QCM transducer and achieved a 17.2–200 ng/mL detection range with less than 10 min analysis time, which is superior to its QCM counterparts and comparable to SPR transducer systems [49].

## 5. Conclusions

OTA is a potent carcinogen and threatens human and animal health. Thus, its presence in food and feed commodities are regulated and monitored. Although OTA contamination is successfully monitored by lab-based methods and devices, there is an increasing demand for on-site monitoring devices. In this work a QCM-based immunobiosensor was developed by direct immobilization of OTA to an aminated sensor surface. Avoiding protein immobilization enabled regeneration with a quite harsh solution, 50 mM NaOH and 1% SDS, and reuse of the sensor surface 13 times without a loss of performance. The developed quartz crystal microbalance (QCM) immunosensor was able to detect OTA in the 17.2–200 ng/mL detection range. The Commission of the European Communities Recommendation (2006/576) guidance values for OTA in feedstuffs are 250 ppb for cereals and cereal products, 50 ppb for feedstuffs for pigs, and 100 ppb for feedstuffs for poultry [57]. Thus, the developed sensor may be used for on-site monitoring of OTA in feedstuff within the limits and contribute to efficient screening of OTA by reducing the workload and dependence on laboratory-based methods. Further studies will enable the use of this sensor for food analysis by the addition of a pre-concentration step using immunoaffinity columns.

## Figures and Tables

**Table 1:** Different OTA immobilization conditions.

	EDC:OTA	Ethanol (%)	OTA (mM)
Surface 1	20	66%	6.6
Surface 2	40	50%	5
Surface 3	40	25%	2.5

**Table 2:** R^2^, LOD and LOQ values of the developed OTA sensor at different time points.

	R^2^	LOD	LOQ
5 min	0.997	19.3 ng/mL	58.5 ng/mL
10 min	0.998	17.2 ng/mL	52.0 ng/mL
Saturation	0.995	26.5 ng/mL	80.2 ng/mL

## Supplementary Materials

The following are available online at http://www.mdpi.com/1424-8220/18/4/1161/s1, Figure S1: The surface prepared by immobilization of OTA-cBSA lost its capability to bind 10F4 antibody upon regeneration with 50 mM NaOH and 1% SDS.
