# Supplementary material for: Label-Free QCM Immunosensor for the Detection of Ochratoxin A
Source: Sensors (Basel). 2018 Apr 11;18(4):1161. doi: 10.3390/s18041161 (PMC5948555; doi:10.3390/s18041161)
Supplement: Supplementary file 1 [file sensors-18-01161-s001.pdf]

Supplementary 1.

Although not presented in this manuscript, at the beginning of this study we were also concerned about accessibility of the analyte when directly immobilized. Therefore we designed an experiment to compare the measurement efficiencies of OTA-BSA immobilized and OTA immobilized sensors. In immobilization, every step was the same except the analyte (OTA/OTA-BSA) added in the reaction. In this experiment it was seen that although the results were comparable at the beginning, the surface prepared by immobilization of OTA-BSA quickly lost its efficiency upon regeneration.

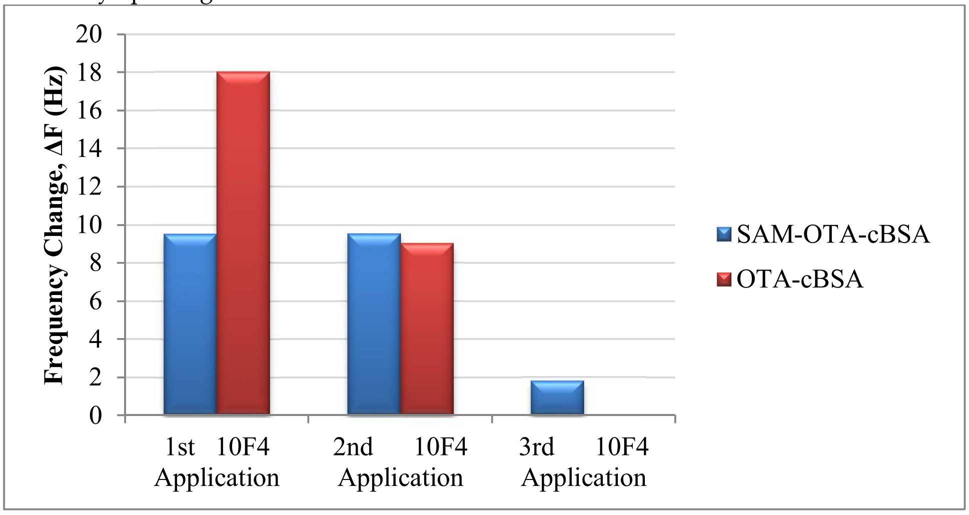

Figure Supplement 1: The surface prepared by immobilization of OTA-cBSA lost its capability to bind 10F4 antibody upon regeneration with 50 mM NaOH and 1% SDS.
